# Clinical Changes and Uterine Hemodynamic in Pyometra Medically Treated Bitches

**DOI:** 10.3390/ani10112011

**Published:** 2020-10-31

**Authors:** Roberto Rodrigues da Rosa Filho, Maíra Morales Brito, Thaís Gomes Faustino, Leticia Lima de Almeida, Thayná Pantoja Gardés, Roberta Ferreira Leite, Camila Infantosi Vannucchi

**Affiliations:** Department of Animal Reproduction, School of Veterinary Medicine and Animal Science, University of São Paulo, São Paulo 05508-270, Brazil; betorrf@gmail.com (R.R.d.R.F.); maira.brito@usp.br (M.M.B.); thais.faustino@usp.br (T.G.F.); l.almeida@usp.br (L.L.d.A.); thay.gardes@gmail.com (T.P.G.); robertaleite@usp.br (R.F.L.)

**Keywords:** aglepristone, prostaglandin, Doppler, uterus

## Abstract

**Simple Summary:**

Pyometra is an infectious uterine disorder marked by important clinical alterations and uterine contamination and blood flow changes in bitches. Although uterine removal is the most common mode of treatment, conservative therapy is an alternative. However, the success of medical treatment in restoring both clinical and uterine alterations has not yet been attested. Thus, we aimed to analyze the effects of both aglepristone alone or in combination with prostaglandin (associative) on clinical, laboratory and uterine hemodynamic features. Bitches were clinically and ultrasonographically (uterine blood flow) evaluated and medically followed-up throughout treatment (8 days) and castrated for local uterine analysis after 9 days of therapy onset. Associative therapy led to altered respiratory rate and decreased body temperature. Both treatments resolved bitches’ clinical condition and laboratory changes, and reduced uterine size. However, associative protocol was more effective in decreasing uterine vascularization and modulating uterine blood flow. Nevertheless, uterus remained microscopically altered soon after both therapy ending, thus not fully restoring local uterine changes in a short-term evaluation.

**Abstract:**

Cystic endometrial hyperplasia–pyometra complex is a canine endometrial disorder, considered the most common and important among uterine diseases. The treatment of choice is ovariohysterectomy, but medical treatments have become an alternative. However, no studies have been performed in order to evaluate uterine hemodynamic changes during medical treatment for pyometra bitches. Therefore, the aim of this study was to compare the effectiveness of two medical protocols by means of clinical (heart rate, respiratory rate and body temperature), medullar response (complete blood count (CBC)) and uterine hemodynamic evaluation in pyometra bitches. For such purpose, 10 bitches with pyometra were assigned to two groups: Aglepristone Group (n = 5) and Associative Group (aglepristone + prostaglandin; n = 5). The Associative Group had altered respiratory rate and decreased body temperature. The Aglepristone Group had decreased plasma protein, however, leukocyte count reduced over time for both therapeutic protocols. Uterine area and vascularization score decreased throughout treatment. There was a significant reduction in uterine perimeter, area and vascularization score. The Associative Group had lower final diastolic velocity and higher systole:diastole ratio (S/D), pulsatility index (PI) and resistance index (RI). In conclusion, both medical treatments are effective in reversing clinical and CBC changes of pyometra, especially aglepristone, however they cannot fully restore local uterine changes in a short-term evaluation. Conversely, associative therapy was more effective in decreasing uterine vascularization and modulating uterine blood flow.

## 1. Introduction

Genitourinary disorders account for approximately 16–18% of all surgical pathologies in small animal veterinary hospitals and can be considered the second most frequent surgical cases [1]. Among the uterine disorders, the complex Cystic Endometrial Hyperplasia–Pyometra (CEH–Pyometra) is the most frequent and important endometrial disorder in non-castrated bitches [2]. About 25% of all bitches are diagnosed with pyometra before 10 years of age and the risk of developing pyometra exceeds 50% in certain dog breeds [3].

CEH–Pyometra is defined as an uterine inflammatory disorder in which thickening of the endometrial wall (CEH) coupled with bacterial infection lead to purulent content within the uterine lumen during or soon after the period of high blood progesterone concentration [4]. Although pyometra starts locally at uterine level, local changes wind up leading to important systemic illness, affecting multiple organs [5], and thus can be considered a medical emergency [6]. More recently, pyometra has been related to increased uterine blood flow and decreased vascular resistance, along with inflammatory, cell proliferation and local angiogenesis [7,8]. Based on such novel uterine hemodynamic data, Doppler ultrasonography can be used as a diagnostic tool for pyometra in bitches. As a non-invasive test, it provides important information on blood flow through the uterine artery, as well as tissue perfusion, blood vessel architecture and hemodynamic features [9].

If left untreated, pyometra mortality rate achieves 3–10% [10,11]. Ovariohysterectomy is the treatment of choice for bitches with closed cervix pyometra or remarkable systemic changes [12,13]. However, several medical treatments for breeding bitches have arose over the years, also recommended for unfavorable clinical conditions for anesthesia or severe compromised senile bitches [14]. For the medical treatment of pyometra, natural or synthetic prostaglandin F2-α (PGF2-α) is an option in young bitches with no kidney and liver dysfunction, by means of luteolysis and uterine contraction [14,15]. Nevertheless, important side effects are registered, as, for example, hypothermia, diarrhea, hypersalivation or vomiting, depression or excitation with shivering [14]. Thus, in order to reduce the occurrence of undesirable effects, low doses of PGF2-α can be associated with a prolactine-inhibiting drug (cabergoline) [16,17], antiprogestin (aglepristone) [12] or GnRH antagonists (acyline) [18]. Aglepristone is a competitive progesterone antagonist, thus inhibiting the hormone-related changes [14,15,19,20,21], however it is contraindicated in bitches with liver and kidney dysfunction, diabetes and suprarenal insufficiency, besides not being available world-wide [12,22]. The effectiveness of pyometra therapy solely with aglepristone has been previously attested, however, the combination with other drugs can enhance the successful rate of treatment. Thus, we hypothesize that the combined action of both aglepristone and prostaglandin (myometrial contractility and cervical opening) is more efficient in promoting discharge of uterine content, and thus more rapidly reversing pyometra clinical signs and uterine changes rather than aglepristone alone.

Although the success of medical treatment from a clinical point of view (clinical signs, bone marrow activity, and discharge of uterine content) has already been suggested [12,21,22], no studies on uterine hemodynamical changes have been undertaken, with special reference to an overtime evolution as a possible prognostic or follow-up variable. Therefore, the aim of this study was to compare the effectiveness of two medical protocols to treat pyometra bitches by means of clinical evaluation, medullar response, uterine hemodynamic and vascularization analysis throughout treatment.

## 2. Materials and Methods

### 2.1. Ethical Approval

The current study was approved by the Bioethics Committee of the School of Veterinary Medicine and Animal Science-University of São Paulo, under protocol 5997311017.

### 2.2. Animals and Study Design

The study was a prospective randomized trial with 10 pyometra bitches, aged 4 to 10 years, of different breeds and body weights (Table 1). All bitches were nulliparous and within the diestrus phase of the estrus cycle, identified by means of vaginal cytology performed at the time of pyometra diagnosis.

All bitches were diagnosed with open-cervix pyometra by means of clinical signs, physical examination, laboratory findings, coupled with uterine ultrasonographic exam: intraluminal uterine content with high endometrial vascularization, increased uterine blood flow and decreased resistance of the uterine artery were assessed [8]. The main observed clinical signs were sanguinopurulent or purulent vaginal discharge, lethargy, inappetance, depression and dehydration. Bitches with severe systemic conditions (kidney and liver dysfunction; sepsis or peritonitis) or closed cervix pyometra were considered as exclusion criteria. All bitches had elevated white blood cell count, anemia, hyperproteinemia, whereas blood urea nitrogen and creatinine were within the normal range for dogs.

The bitches were randomly assigned to two experimental groups, according to the therapeutic protocol:

- Aglepristone (RU46534) Group (n = 5): bitches subjected to daily subcutaneous injections of 10 mg/Kg of aglepristone (Alizin^®^-30 mg/100 mL-VIRBAC, Carros, France) on days 1, 2 and 8 [12];
- Associative Therapy Group (n = 5) bitches subjected to daily subcutaneous injections of 10 mg/Kg of aglepristone (Alizin^®^-30 mg/100 mL-VIRBAC, Carros, France) on days 1, 2 and 8 [12], coupled with daily intramuscular injections of 1 µg/kg of cloprostenol (Ovolute^®^-7,5 mg/100 mL-DRAG PHARMA, Santiago, Chile) from days 1 to 7 (protocol adapted from [12]).

To assure the appropriate sample size, an analysis was conducted with the SAS Power and Sample Size 12 (SAS Institute Inc., Cary, NC, USA). A retrospective analysis of the data indicated there was a power of 0.97, which is considered an acceptable statistical power (at least 0.8). Hence, a minimum of five dogs per group were sufficient to demonstrate significant differences in the data.

All bitches remained under hospitalization and intensive care, within controlled environment. Support therapy (5–10 mL/kg/h of intravenous 0.9% saline solution, daily) and broad-spectrum antibiotic therapy (10 mg/Kg/day of enrofloxacin (Enromic^®^-5 mg/100 mL-Microsules Laboratory-Uruguay) and 30 mg/Kg/day of metronidazole (Endonidazol^®^-5 mg/mL-Fresenius Kabi Brazil Ltd.a, Aquiraz, Ceara)) were provided from days 1 to 8.

At the 9th day after pyometra diagnosis and therapeutic protocol, bitches were ovariohysterectomized and uterine fragments were subjected to histopathological examination.

### 2.3. Clinical, Hematological and Nitric Oxide Evaluation

Clinical evaluation was performed only on day 1 (at the moment of diagnosis and onset of treatment) and day 2, referring to the only days in which both drugs (aglepristone and cloprostenol) were administered to each bitch. Clinical examination, consisting of heart rate (HR, bpm), respiratory rate (RR, mpm) and body temperature (°C), was always done at the following timepoints: before treatment, 15 and 60 min post-therapy and by the same investigator. In addition, all possible side effects were recorded, such as vomiting, diarrhea, salivation and mydriasis.

Blood samples were collected following asepsis by puncturing the right or left jugular vein on days 1, 4 and 8 after the onset of treatment and immediately transferred to tubes with EDTA anticoagulant for red blood cell count (RBC, 10^6^/mm³), hematocrit (PCV, %), leukocyte count (mm³) and plasma protein (g/dL). Hematimetric variables were measured through an automated hematologic analyzer (ADVIA 2120i Hematology System, Siemens ^®^, Munich, Germany).

For serum nitric oxide (NO) assessment, blood samples were allowed to clot at room temperature into tubes without anticoagulant and centrifuged for 10 min at 1500 X g. Serum was drawn off, separated in aliquots and stored at −20 °C until it was analyzed. Nitric oxide levels were indirectly determined according to the protocol of Maurer et al. [23]. Deproteinization process was performed by adding 20 μL of 30% zinc sulphate (diluted in 1N hydrochloric acid) to 400 μL of serum. Mixture was then centrifuged at 13000 rpm for 15 min and the supernatant was separated. Subsequently, 50 μL of the deproteinized sample and 50 μL of vanadium chloride (8 mg/mL, diluted in 1N hydrochloric acid) were added to a microplate, which was kept in the absence of light until quantification. For the Griess reaction, 25 μL of 2% sulfanilamide was added, diluted in 5% phosphoric acid and incubated for 10 min. Then, 25 μL of 0.2% *N*-(1-naphthyl) ethylenediamine (diluted in deionized water) was added and the sample was incubated at 37 °C for 20 min. Nitric oxide was quantified spectrophotometrically at a wavelength of 540 nm (Biotek EL808^®^, Winooski, VT, USA) and results were described as μmol/L.

### 2.4. B-Mode, Color-Flow Doppler and Pulsed-Wave Doppler Procedures

Transabdominal ultrasound evaluation was performed always by the same operator, with the use of a 5 MHz micro convex transducer (Mindray^®^ ultrasound M5Vet, Shenzhen, China) in a right and left lateral recumbence. Ultrasonographic examination was carried out daily during the 8 days of treatment, always before the therapeutic protocol. Transversal cross-sections of the uterine horns were made in B-mode as an ellipse, and the area was calculated by the following formula: *R1 x R2 x* *π*, considering *R1* = uterine width/2 and *R2* = uterine height/2 [24]. We always considered the uterine cross-section with the higher dimension for our data collection purpose.

For the qualitative evaluation of the endometrial vasculature, scanning of uterine horns was preferably carried out in B-mode cross-section, followed by Color flow Doppler. A score from 1 to 3 was adopted, considering score 1 as the minimum degree of endometrial vasculature and score 3, the maximum degree, according to classification proposed by Veiga et al. [8].

Dopplervelocimetry of the right and left uterine arteries were performed in longitudinal section at the lateral region of the uterine body, using the bladder as an acoustic window. Pulsed-wave Doppler was used to characterize the waveform and the following vascular parameters were measured: blood flow velocity (peak systolic velocity (PS), end diastolic velocity (ED) and time average maximum velocity (TAMAX))and hemodynamic indexes (resistance index (RI), pulsatility index (PI) and peak systolic: diastolic velocity (S/D)). Resistance index of the uterine artery was also used to classify the uterine horns as the *most compromised* and *less compromised*, i.e., the uterine horn containing the artery with the lowest RI value was considered the *most compromised* and, conversely, the contralateral uterine horn was classified as the *less compromised* [8].

### 2.5. Macroscopic and Histological Uterine Examination

Immediately after hysterectomy, uteri were evaluated macroscopically for the extent of uterine alterations (superficial and focal changes, general aspect of the endometrium, uterine morphology, presence of endometrial cysts and hyperplasia). Subsequently, uterine tissue samples were obtained (0.5 cm), preferably collected always from the same uterine region. Fragments were washed with 0.9% saline solution and fixed in 10% buffered formaldehyde solution for at least 48 h, and subsequently stored in 70% alcohol solution for later inclusion in paraffin, dehydration, and diaphanization. Sections (5-μm) were randomly selected and submitted to deparaffinization and stained with hematoxylin-eosin [8]. At least 10 random fields of view from each uterine section were analyzed using OLYMPUS BX60^®^ microscope, Tokyo, Japan with a Zeiss AxioCam HRc^®^ digital camera, Jena, Germany, equipped with a 20*x* objective lens using Zen Blue 2.6^®^ *software* (Carl Zeiss, Jena, Germany). A descriptive evaluation of the main histological findings was performed.

### 2.6. Statistical Analysis

All data were evaluated using the SAS System for Windows version 9.3 (SAS Institute Inc., Cary, NC, USA). Differences between groups were analyzed using parametric tests, according to the residual normality (Gaussian distribution) and variance homogeneity. Whenever one of these assumptions was not valid, data were transformed, following transformations suggested and performed by the Guided Data Analysis Procedure of the SAS System for Windows.

The effects of groups and time of evaluation and interactions between them were estimated by the repeated measures analysis of variance (Mixed Procedure of SAS). Significant interactions were considered if *p* < 0.1, which is considered an adequate interaction p-value for factorial experiments [25]. For the clinical examination, no triple interaction (Group (aglepristone vs. associative) *x* Moment (0, 15 and 60 min) *x* time-points (days 1 and 2)) was observed, thus the following interactions were considered: Group *x* Time, Group *x* Moment and Time *x* Moment. If no significant interactions occurred, Student t-test was used for comparison between Groups (combining all times and moments) and between time (combining all groups and moments). In order to compare the time-points (combining all groups and times), the Least Significant Difference (LSD) test was used.

For the hematological variables, nitric oxide blood concentration, uterine area and qualitative evaluation of the endometrial vasculature, data were evaluated according to a double interaction: groups (aglepristone vs. associative) and time-points (days 1 to 8). If no significant interactions occurred, the effect of groups was analyzed combining all time-points by the Student *t*-test and conversely, time-points were compared combing all groups through the LSD test.

Considering the Power Doppler variables, no triple interaction (Groups (aglepristone vs. associative), degree (most compromised vs. less compromised) and time-points (days 1 to 8)) was observed, thus the following interactions were considered: Group *x* Degree, Group *x* Time and Degree *x* Time. If no significant interactions occurred, Student *t*-test was used to compare between groups (combining all time-points and degrees) and degrees (combining all time-points and groups); and the LSD test was used to compare time-points (combining all degrees and groups).

Variables were also submitted to Pearson correlation analysis, considering only the group effect. Results are reported as untransformed means ± SE and statistical differences were considered significant if *p* ≤ 0.05.

## 3. Results

No triple interactions were observed in this experiment. However, there was a significant interaction between groups and moments post-therapy for RR (*p* = 0.04) and between groups and time-points for plasma protein (*p* = 0.03) and uterine area (*p* = 0.08).

### 3.1. Clinical, Hematological and Nitric Oxide evaluation

Bitches of the Associative Group had a significant increase (*p* = 0.0004) in respiratory rate between 0 and 15 min post-therapy (37 ± 4.3 and 156 ± 22.2 mpm, respectively), followed by a decrease at 60 min after treatment (65 ± 17.0 mpm). Furthermore, RR was higher (*p* = 0.004) in the Associative Group (156 ± 22.2 mpm) compared to the Aglepristone Group (57 ± 17.5 mpm) at 15 min post-therapy. Associative therapy led to a significant decrease (*p* = 0.04) in body temperature between day 1 (38.11 ± 0.11 °C) and day 2 (37.77 ± 0.13 °C) of treatment. No statistical difference (*p* > 0.05) between groups was observed for HR and body temperature.

Regarding the occurrence of side effects, bitches of the Associative Group had short-term side effects (emesis, sialorrhea, diarrhea, mydriasis or miosis) after PGF-2α injection, mostly between 15 and 60 min post-therapy. Conversely, the Aglepristone Group presented no short-term side effects.

There was a significant decrease (*p* = 0.05) in plasma protein between day 1 (8.68 ± 0.66 g/dL) and day 4 (6.85 ± 0.22 g/dL) after the onset of aglepristone therapy, but not for the Associative Group. A progressive decrease (*p* = 0.04) in leukocyte count occurred during the course of therapy, with significant difference between day 1 (37,963 ± 4325.29 mm^3^) and day 8 (22,640 ± 3638.79 mm^3^), regardless of the therapeutic protocol. No significant difference (*p* > 0.05) was observed between groups for plasma protein and leukocyte count. Bitches of the Associative Group had lower RBC (*p* = 0.04) (4.59 ± 0.20 *x* 10^6^/mm^3^) and PCV (*p* = 0.02) (31.37 ± 1.35%) compared to the Aglepristone Group (5.57 ± 0.38 *x* 10^6^/mm^3^ and 37.29 ± 2.09%, respectively). Conversely, the Associative Group had higher (*p* = 0.03) NO concentration (2.36 ± 0.04 μmol/L) compared to the Aglepristone Group (2.21 ± 0.05 μmol/L), regardless of the time-point. No difference in NO serum concentration was observed over time.

### 3.2. Uterine Ultrassonographic Examinations

**Figure 1:**
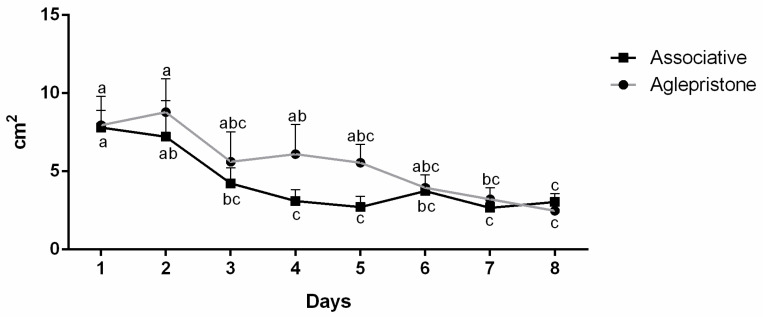
Uterine area (cm²) throughout the experimental period (1–8 days) in the Associative and Aglepristone groups. ^a–c^ values with different letters differ significantly with time.

Although a progressive decrease (*p* < 0.05) in uterine area occurred throughout the experimental period (Figure 1), no statistical difference (*p* > 0.05) between groups was observed. The Aglepristone Group had a significant decrease (*p* = 0.05) in uterine area between day 1 and day 8, whereas in the Associative Group, a significant decrease (*p* = 0.006) in uterine area occurred after day 3 of therapy (Figure 1).

**Figure 2:**
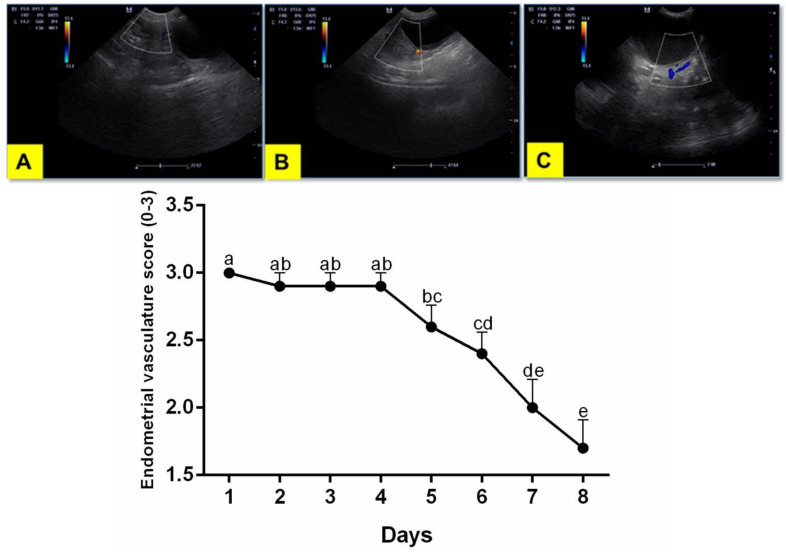
Endometrial vascularization score in cross-section Color Flow Doppler. (**A**) score 1 (minimum), (**B**) score 2 (intermediary) and (**C**) score 3 (maximum). **(Below)** Endometrial vascularization score (1–3) throughout the experimental period (1–8 days). ^a–e^ values with different letters differ significantly with time.

Regarding endometrial vascularization, there was a decrease throughout treatment, with a significant difference (*p* < 0.0001) between day 1 and days 5-8, regardless of the therapeutic protocol (Figure 2).

**Figure 3:**
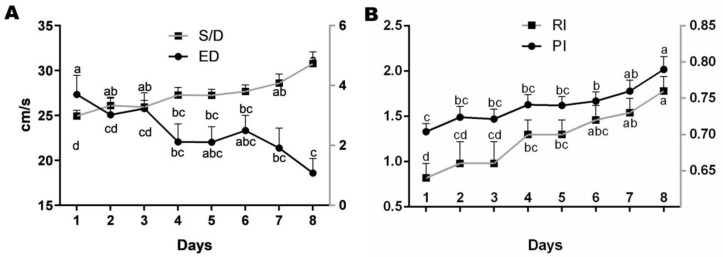
(**A**) End diastolic blood velocity (ED; cm/s) and Peak systolic: diastolic velocity (S/D) of the uterine artery throughout the experimental period. (**B**) Pulsatility index (PI) and resistance index (RI) of the uterine artery throughout the experimental period. ^a–c^ values with different letters differ significantly with time.

We verified a progressive decrease in ED of the uterine artery over time, with statistical difference (*p* = 0.03) among day 1 and days 4, 7 and 8, regardless of the therapeutic protocol (Figure 3A). For the hemodynamics indexes of the uterine artery, there was a progressive increase along therapy, with statistical difference (*p* = 0.0003) between day 1 and day 4 onwards for the S/D, regardless of the treatment protocol (Figure 3A). In addition, we observed statistical difference (*p* = 0.0005) among day 1 of therapy and day 4 onwards for the RI of the uterine artery, whereas for the PI, there was statistical difference (*p* = 0.001) among day 1 and day 6 onwards (Figure 3B). No difference in the PS and TAMAX of the uterine artery occurred throughout the experimental period.

**Figure 4:**
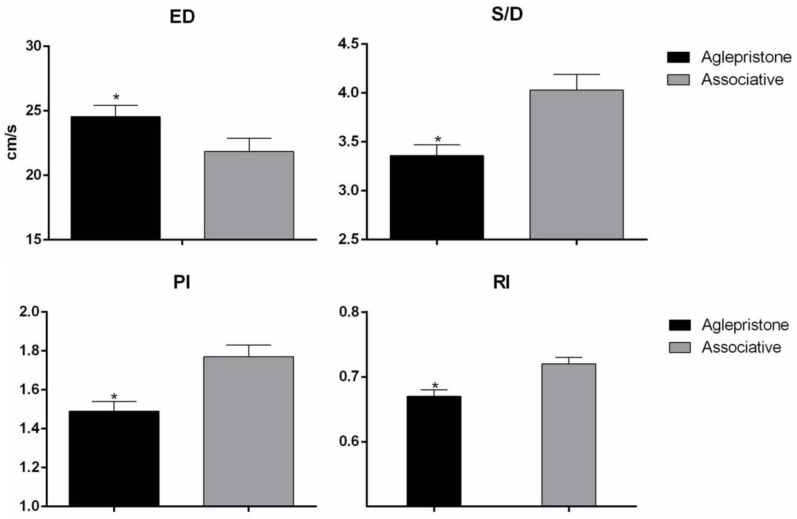
End diastolic blood velocity (ED; cm/s), peak systolic: diastolic velocity (S/D), pulsatility index (PI) and resistance index (RI) of the artery uterine in the Aglepristone and associative groups. * indicate significant differences between groups (*p* < 0.05).

Comparing the experimental groups, the Associative Group had a lower (*p* = 0.02) ED and higher (*p* = 0.0007) S/D in relation to the Aglepristone Group (Figure 4). Conversely, the Associative Group had higher RI and PI of the uterine artery (*p* = 0.0009 and *p* = 0.0006, respectively) compared to the Aglepristone Group (Figure 4). No statistical difference (*p* > 0.05) between degrees of uterine artery alteration (most and less compromised) was verified for the dopplervelocimetry variables.

### 3.3. Macroscopic and Histological Uterine Examination

Macroscopically, we observed, equally in both experimental groups, reduction in uterine thickness and simetrical morphology of uterine horns, in comparison to pyometra bitches subjected to immediate hysterectomy after diagnosis (data not presented). However, mild endometrial hyperplasia still remained. On the other hand, no endometrial cysts and purulent or sanguinopurulent intra-luminal content were observed in both groups.

**Figure 5:**
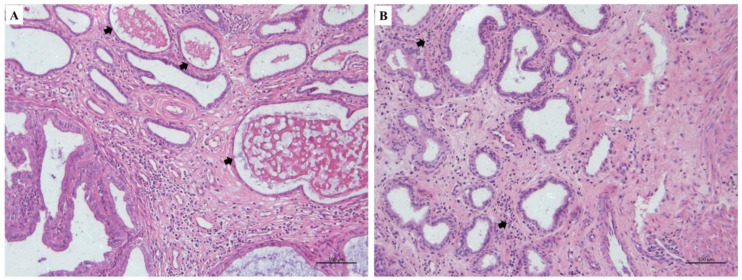
Microscopic uterine findings after pyometra therapeutic protocol. (**A**) endometrial cysts variables in size (arrows) throughout the endometrium; (**B**) stromal content of polymorphonucleated cells (neutrophils) and inflammatory cells infiltrate (arrows). Hematoxylin and eosin stain. Bar scale: 100 μm.

Uterine histological analysis revealed distinct sized cysts throughout the endometrium (Figure 5A), as well as endometrial edema and hyperplasia in both experimental groups. There was an increased number of endometrial glands, stromal inflammatory cells (polymorphonucleated cells), focal hemorrhage and vascular congestion (Figure 5B).

### 3.4. Correlations Analysis

For the Aglepristone Group, negative correlations were observed between uterine area and PI (r = −0.32; *p* = 0.04), RI (r = −0.33; *p* = 0.03) and S/D (r = −0.31; *p* = 0.04) of the uterine artery. Additionally, leukocyte count correlated negatively with PI (r = −0.71; *p* = 0.002), RI (r = −0.69; *p* = 0.004) and S/D (r = −0.65; *p* = 0.008) of the uterine artery.

For the Associative Group, uterine area correlated positively with ED of the uterine artery (r = 0.33; *p* = 0.04) and negatively with PI (r = −0.42; *p* = 0.006), RI (r = −0.44; *p* = 0.003) and S/D (r = −0.42; *p* = 0.006) of the uterine artery. Additionally, leukocyte count correlated positively with ED (r = 0.77; *p* = 0.001) and TAMAX (r = 0.74; *p* = 0.002); and negatively with PI (r = −0.82; *p* = 0.0003), RI (r = −0.81; *p* = 0.0004) and S/D (r = −0.81; *p* = 0.0004). Blood NO concentration correlated positively with PI (r = 0.40; *p* = 0.009), RI (r = 0.38; *p* = 0.02) and S/D (r = 0.39; *p* = 0.01) of the uterine artery.

## 4. Discussion

The present study aimed to evaluate possible changes in uterine vascularization and hemodynamic, as well as clinical assessment of bitches submitted to medical treatment for pyometra.

Similar to a previous report in dogs [12], we also observed short-term side effects of PGF2-α throughout the associative therapy protocol, although we employed a rather low dose of prostaglandin (1 µg/kg). Bitches of the Associative Group had significant increase in respiratory rate after 15 min of PGF2-α injection, possibly due to a bronchoconstriction effect [26,27]. However, tachypnea was a short-term effect that lasted for 60 min after PGF2-α administration. Indeed, Wasserman [28] demonstrated that intravenous injection of PGF2-α causes respiratory effects after 1 min, persisting for 10 min. In our study, the use of the intramuscular route delayed the respiratory side effect compared to the intravenous injection. Unlike the report of Jena et al. [15], hypersalivation was the most frequent adverse effect of PGF2α in the present study. Although several side effects were reported resulting from the PGF2α action on smooth muscles, such as salivation, vomiting and diarrhea [12], it is important to point out that the frequency of undesirable secondary effects subsided throughout therapy, suggesting a progressive clinical PGF2α tolerance. Even though hypothermia has been reported after the injection of PGF2α [14], bitches’ body temperature remained within the reference range after treatment. On the other hand, there was a significant decrease in body temperature between the first and second days of therapy, regardless of the medical treatment. Thus, we assume that the thermogenic action of progesterone was blocked by the use of luteolytic (PGF2α) and antiprogestagen (aglepristone) drugs, and was not a direct effect of the therapeutic protocol.

At the onset of therapy, plasma protein concentrations of pyometra-treated bitches were above the reference range, suggesting hyperproteinemia. Frequently of inflammatory origin [29], the increase in circulating proteins can be associated with the uterine inflammation [5]. On the other hand, the Aglepristone Group had a significant decrease in plasma protein concentrations at day 4 of treatment, reaching normoproteinemia, thus suggesting an ongoing recovery of the uterine inflammatory process. Interestingly, no difference in protein concentrations was verified over time for the Associative Group, retaining the hyperproteinemic status. Moreover, the red blood cell count and hematocrit remained within the reference range throughout the aglepristone therapy, rather significantly higher compared to the Associative Group, which presented anemia (deficient erythropoiesis). We assume that such findings (hyperproteinemia and non-regenerative anemia) are a consequence of an acute inflammatory response of daily injections of PGF2α [30] and should not be considered a follow-up parameter for pyometra treatment when prostaglandin protocol is applied. For such a goal, it is advisable to analyze blood leukocyte count. Regardless of the experimental group, we observed a significant decrease in leukocyte count between day 1 and 8 of treatment, suggesting the effectiveness of uterine treatment, coupled by an efficient antibiotic therapy.

During the entire extent of the experimental period, it was possible to observe discharge of uterine content in both treatment protocols, leading to a significant decrease in uterine area. Although Fieni (2006) [12] observed a reduction in uterine diameter only after 8 days of pyometra treatment with both aglepristone and associative protocol (starting prostaglandin injections at day 3), in our study, a significant decrease in uterine area occurred earlier in the Associative Group (3th day of treatment) compared to the Aglepristone Group (7th day of treatment). Thus, despite the daily injections of cloprostenol, this result can be a differential of the associative protocol, by a sum action of PGF2α and aglepristone in promoting myometrial contractions, and thus removing uterine content. Nevertheless, future studies should be performed, aiming to reduce the frequency of prostaglandin injections in order to reduce unpleasant side-effects, yet efficiently discharge uterine content.

Pyometra is associated with an increase in endometrial vascularization and blood flow of the uterine artery, compared to non-affected uterus, as well as a decrease in vascular resistance [7,8]. In our study, a progressive decrease in endometrial vascularization occurred throughout the experimental period, suggesting a positive effect of treatment. On the other hand, despite the significant reduction in uterine vascularization at day 8 of treatment, it did not reach the expected value for diestrous bitches. Therefore, we believe that endometrial vascularization during pyometra treatment requires a long-term return to normal conditions, demanding a posteriori evaluation in order to attest therapeutic efficacy. Regarding uterine artery blood flow, we observed a progressive decrease and increase, respectively, in the end diastolic velocity (ED) and peak systolic: diastolic velocity (S/D), suggesting significant hemodynamic changes throughout treatment. In addition, leukocyte count negatively correlated with hemodynamic indexes (PI, RI and S/D) and positively correlated with the end diastolic velocity (ED) of the uterine artery. These data reinforce the beneficial influence of pyometra treatment protocols on uterine blood flow and uterine infection control. It is important to point out that progesterone has a vasodilator action [31], by modulating the synthesis of blood vessels’ nitric oxide and, thus, decreasing Ca^2+^ concentration of vascular smooth muscle cells [32]. Hence, progesterone blockage or luteolysis by the action of aglepristone and prostaglandin, respectively, coupled with the reduction in inflammation led to the decrease in uterine artery blood flow. We observed an increase in the resistance and pulsatility indexes of the uterine artery throughout pyometra treatment, denoting a progressive increase in vascular resistance, ultimately corroborating a positive and effective response to medical therapy.

Comparing the experimental groups, the Associative Group had lower end diastolic velocity (ED) and higher hemodynamic indexes (PI, RI and S/D) of the uterine artery, in comparison to the Aglepristone Group. Therefore, we can infer that the associative protocol promoted more significant effects on uterine blood flow and uterine artery resistance. These findings suggest an additional overall hemodynamic action of prostaglandin, by leading to vasoconstriction [33] and moderate systemic hypertension [34], ultimately increasing peripheral resistance [35] and decreasing peripheral blood flow [36]. Thus, we can infer that the hemodynamics changes in the uterine artery with the use of the associative therapy are also a consequence of uterine inflammation overcome. Moreover, we observed a positive effect of prostaglandin in respect to uterine artery hemodynamic recovery, mainly when vascular features are compared to untreated pyometra bitches [8].

Interestingly, we observed a positive correlation between hemodynamics indexes of the uterine artery and serum NO in the Associative Group. In addition, the Associative Group had higher serum NO concentrations than the Aglepristone Group, regardless of treatment time-point. We assume that the local vasoconstrictor effect of daily prostaglandin injection led to reflexive increase in systemic NO concentrations, thus increasing general blood flow as a way of compensating for a possible vascular counterpart between uterine and systemic circulation. NO is a gaseous state molecule with free passage through cell membrane into cytoplasm. NO has a modulatory role in vascular permeability, by stimulating cyclic guanosine monophosphate (GMPc) synthesis, thus increasing cellular calcium influx, ultimately, promoting smooth muscle relaxation and adjustment of vascular diameter and resistance [37,38]. Additionally, nitric oxide is considered a potent antioxidant [39] and an oxidative stress marker in pyometra mares [40]. Hence, these data support the positive effect of the associative treatment by also mobilizing the NO antioxidant effect and reducing systemic oxidative stress in pyometra bitches.

Although uterine histological exam is the main tool for pyometra definitive diagnosis [8,41,42], to the author’s knowledge, no such analysis has been previously performed after pyometra medical therapy in bitches. The uterine lesions found in the present study after treatment resembled class I and II of the pyometra classification of Dow [43] and moderate CEH and endometritis of De Bosschere [44] classification. Taking together our results, we suggest that both medical treatments can successfully resolve pyometra clinical condition, without fully restoring local uterine changes in a short-term evaluation. Nevertheless, future studies should be performed with a further accurate quantitative histological analysis, as well as taking into consideration a more prolonged period from the end of therapy and uterine microscopic evaluation.

In the present study, no hemodynamic difference occurred between the *most* and *least compromised* uterine artery throughout pyometra treatment. Thus, we propose that the follow-up of uterine blood flow by ultrasound during medical treatment can be performed irrespective of the uterine artery localization, thus making the diagnostic approach easier.

## 5. Conclusions

In conclusion, pyometra medical treatment is a feasible therapeutic measure in bitches, irrespective of the protocol (aglepristone alone or in association with PGF2-α). Nevertheless, the associative therapy can significantly reverse pyometra hematological changes, in addition to an efficient decrease in endometrial vascularization, through an adjustment of uterine artery blood flow in bitches.

To our knowledge, this is the first report to assess uterine vascularization during pyometra medical treatment, however, further studies should be undertaken to more accurately understand the mechanism of action of therapeutic protocols in modulating uterine blood flow.

## Figures and Tables

**Table 1:** Females features (breed, age and weight) in the Aglepristone (n = 5) and Associative (n = 5) groups.

	Breed	Age (yo)	Weight (kg)
Aglepristone Group	Dachshund	8	8.8
	Weimaraner	8	44
	Lhasa Apso	9	3.6
	Mixed-breed	7	11.3
	Mixed-breed	8	22.3
	X ± SD	8 ± 0.7 ^A^	11.3 ± 16 ^a^
Associative Group	Boxer	9	23
	Mixed-breed	10	16.4
	Mixed-breed	10	13.3
	Mixed-breed	4	11.4
	Labrador	9	35
	X ± SD	9 ± 2.5 ^A^	16.4 ± 9.5 ^a^

^A^ Diferrence between groups was not significant (*p* = 0.67). ^a^ Difference between groups was not significant (*p* = 0.82).
